# Exploring Disparities in Pavement Burns: A Comparative Analysis of Housed and Unhoused Burn Patients

**DOI:** 10.3390/ebj6030038

**Published:** 2025-07-01

**Authors:** Henry Krasner, Emma Chevalier, Samantha Chang, David Slattery, Syed Saquib

**Affiliations:** 1 Kirk Kerkorian School of Medicine at UNLV, University of Nevada Las Vegas, Las Vegas, NV 89106, USA; 2 University of Nevada Las Vegas, Las Vegas, NV 89154, USA; 3 School of Medicine, University of Nevada Reno, Reno, NV 89557, USA; samanthachang@med.unr.edu; 4 Department of Emergency Medicine, Kirk Kerkorian School of Medicine at UNLV, University Medical Center of Southern Nevada, Las Vegas, NV 89106, USA; david.slattery@unlv.edu; 5 Department of Surgery, University of California Irvine Medical Center, Irvine, CA 92868, USA; sfsaquib@gmail.com

**Keywords:** pavement burns, burn injuries, burns, homelessness

## Abstract

In some regions, extreme heat can result in pavement temperatures that are high enough to cause severe burn injuries within seconds of skin contact. This risk is elevated for unhoused individuals who may lack adequate clothing and shelter and have susceptibility to other risk factors, including substance use and in turn loss of consciousness. While prior studies have shown worse outcomes for unhoused individuals due to delays in care and higher susceptibility, there is a lack of data on the impact of pavement burns specifically within this population. This single-institution retrospective cohort study aims to explore burn severity and hospital outcomes in housed vs. unhoused patients with pavement burns. The data were analyzed using independent samples *t*-tests and logistic regression when appropriate, with *p* < 0.05 considered statistically significant. A total of 305 individuals met the inclusion/exclusion criteria and comprised the final study cohort, 17.7% of which were unhoused. There was no significant difference in TBSA, survival to discharge, or hospital length of stay between housed and unhoused patients. While unhoused individuals may still be at heightened risk for pavement burns due to exposure to extreme heat and a lack of protective measures, these results may additionally suggest consistent emergency care for patients regardless of housing status. Furthermore, these results highlight the importance of developing targeted outreach and prevention programs and equitable emergency care protocols for vulnerable populations.

## 1. Introduction

**Figure 1:**
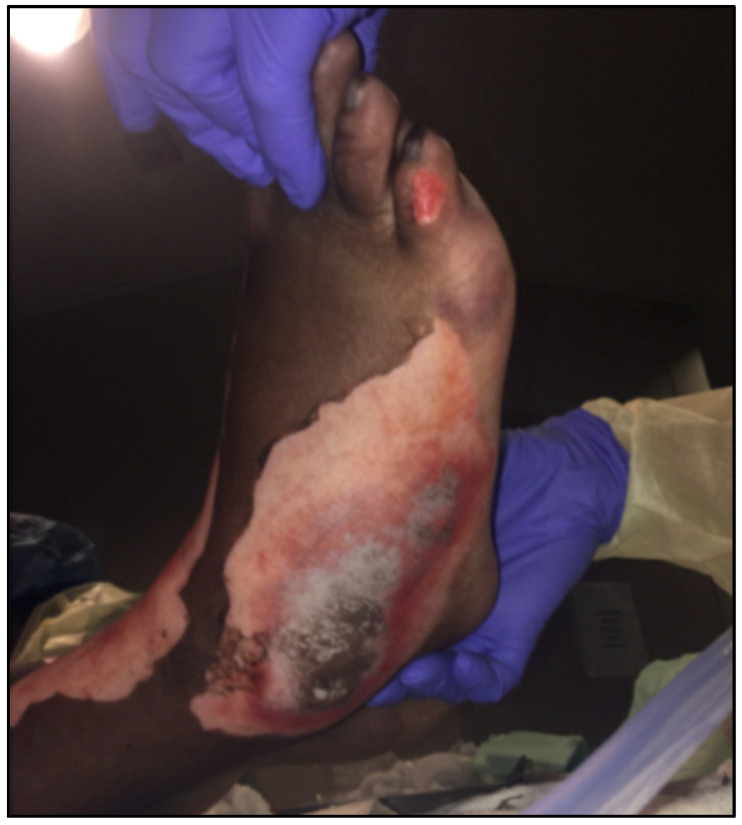
Pavement burn on foot.

In geographic regions such as the Southwestern United States, hot pavements can result in partial- and full-thickness burn injuries within mere seconds of contact with the skin [1]. It has been identified in previous studies that the risk for pavement burn injuries in a desert climate begins at approximately 35 °C (95 °F) and further increases exponentially as the temperature rises [2]. These ambient temperatures are able to produce pavement surface temperatures that are greater than 44 °C (111 °F) [3]. Specifically, one study demonstrated that in Las Vegas, Nevada, asphalt temperatures could climb as high as 74.4 °C (166 °F) during the summer months, well over the threshold required for a pavement burn injury to occur [3]. At these high temperatures, skin barrier proteins become denatured, in turn leading to cell and tissue damage and cutaneous thermal burns [4,5]. Previous studies have indicated that pavement burn patients specifically have been associated with a longer length of hospitalization than patients with other burn injury etiologies [4]. This may be in part due to the fact that pavement burns are uniquely able to convert into a full-thickness burn injury even after an initial partial-thickness burn [4,6]. Additionally, many pavement burn patients experience their injuries at pressure points of the body, which exhibit an especially elevated risk for skin and wound breakdown (Figure 1). Pavement burns often require more surgical intervention on average, including multiple debridement procedures, compared with other etiologies of burn injuries, such as electrical burns. In another study of pavement burn patients in Las Vegas, Nevada, 50.5% of admitted pavement burn patients required burn excision and 35.9% required split-thickness skin grafting in order to manage their extensive injuries [7]. Beyond this, pavement burns can lead to numerous complications, which may result in catastrophic outcomes such as limb amputation or even lead to death in patients with the most severe burn injuries [4,7].

Individuals who are unhoused in the Southwestern United States may have a particularly high risk for pavement burn injuries [8]. A lack of shelter or adequate protective clothing can in turn lead to increased contact time with heated pavements. Furthermore, unhoused populations have been shown to be especially susceptible to known risk factors associated with pavement burn occurrence, including alcohol/drug utilization and disorders that may result in a loss of consciousness [4,6,7,9,10,11]. For example, one 2019 United States study investigated burn patients who utilize methamphetamine, and identified that methamphetamine utilization was associated with lower socioeconomic status (SES) as well as a significantly greater TBSA and longer length of hospital stay in these patients [11]. Some previous studies have demonstrated that burn patients with lower socioeconomic or unhoused status may experience a higher percentage of total body surface area burned (TBSA) than their housed counterparts [12,13]. Other studies have found that unhoused individuals were less likely to undergo burn surgical operations—such as debridement and grafting—than housed patients, which may potentially be due to the implicit bias of healthcare practitioners, placing unhoused burn patients in an especially vulnerable position [14]. Beyond being more likely to be managed nonoperatively for burn injuries, unhoused patients were more likely to receive fewer operations than their housed counterparts when surgery did occur [14]. In regard to pavement burns specifically, in Las Vegas, it was found that unhoused patients frequently presented to a burn center several days or weeks after the occurrence of the initial burn injury due to lack of recognition of the burn severity—which may have further devastating implications on these patients’ outcomes [7]. Notably, the proportion of individuals who are experiencing homelessness in Las Vegas was approximately 24.6 per 10,000 people in 2024—which is substantially higher than the United States national average of 18 per every 10,000 people [13].

Previous studies have demonstrated that unhoused patients may experience worse patient outcomes and more severe burn injuries than their housed counterparts [15,16]. Yet, there is a lack of research on the relationship between housing status and patient experience and outcomes in regard to pavement burns specifically. This study aims to fill this gap by uniquely investigating the impact of pavement burns on unhoused patients in comparison to housed pavement burn patients to determine how the demographic factor of homelessness correlates with these unique burn patients’ experiences and outcomes. We hypothesized that unhoused pavement burn patients would have worse outcomes compared with housed pavement burn patients. Furthermore, we believe that demonstrating the disproportionately harmful impact of pavement burns on unhoused populations may provide evidence that increased public infrastructure to provide shade for unhoused individuals in geographic areas prone to an increased incidence of pavement burns may be beneficial in preventing pavement burn hospitalizations and complications.

## 2. Materials and Methods

### 2.1. Study Design and Setting

This is a single-institution, structured retrospective cohort study of consecutive pavement burn patients admitted to our academic, American Burn Association (ABA)-verified burn center, located in Las Vegas, Nevada from 1 January 2015 to 30 June 2022.

### 2.2. Eligibility Criteria

Adults (age ≥ 18 years of age) that were evaluated and ultimately admitted to the University Medical Center of Southern Nevada Burn Center with a pavement burn injury were included in this study. Patients were excluded from the study if they were under law enforcement custody, pregnant, or had missing primary or secondary outcome data.

### 2.3. Ethical Considerations

This study was approved by our Institutional Review Board on 19 September 2023 (09-05-2023EMPavement) and was determined to involve no more than minimal risk. The data points of the patients analyzed in this study were de-identified and contained no personal identifiers. Furthermore, only the research team had any access to the data utilized for analysis.

### 2.4. Data Collection and Variables

Patients who were able to meet both the inclusion and exclusion criteria were identified using our institution’s burn registry. A trained and monitored research assistant, blinded to the study’s primary and secondary outcomes, abstracted data from the patient’s medical record using a standardized data collection tool and data dictionary [17]. Abstracted data included both patient characteristics and demographics (including housing status, age, sex, and BMI) and clinical variables, such as TBSA, length of hospital stay, and mortality. The primary outcome measure was burn size (% TBSA) in unhoused pavement burn patients compared with housed pavement burn patients. Secondary outcomes included all-cause hospital mortality and hospital length of stay.

### 2.5. Statistical Analysis

Statistical analyses were performed using StataBE (version: STATA18.5). Descriptive statistics were used to summarize the cohort, with categorical variables presented as frequencies and percentages and continuous variables as means ± standard deviations (SDs). Independent samples *t*-tests compared differences in total body surface area (TBSA) and hospital stay duration between housed and unhoused groups. Logistic regression analysis assessed the association between housing status and mortality, adjusting for potential confounders such as age and body mass index (BMI). Odds ratios (ORs) with 95% confidence intervals (CIs) and *p*-values were reported, with statistical significance set at *p* < 0.05. Results are presented in tables with detailed estimates and significance values.

## 3. Results

### 3.1. Demographic Characteristics of Study Cohort

During the study period, there were a total of 319 pavement burn patients who were admitted to the burn center utilized for this study. Ultimately, 305 of these individuals satisfied the inclusion criteria and did not meet any exclusion criteria, thus comprising the final study cohort. Overall, 18.70% of pavement burns were in unhoused *n* = 54 vs. housed *n* = 251 patients. In regard to looking at the baseline characteristics of the study cohort, there was no significant difference in age (mean age unhoused = 51.2 vs. housed = 54.6; 95% CI = (−1.69,8.49) *p* = 0.14) between patients. There was a statistically significant difference in sex between patient groups (95% CI = (1.27–11.32); *p* = 0.0019)). In addition, unhoused patients were found to have a slightly lower mean BMI compared with their housed counterparts (unhoused = 24.84 vs. housed = 28.8, 95% CI = (1.06,1.22); *p* < 0.0001). The demographic characteristics of the study cohort including housing status, age, sex, and BMI are further illustrated in Table 1.

### 3.2. Burn Characteristics and Hospital Course of Unhoused vs. Housed Patients

**Figure 2:**
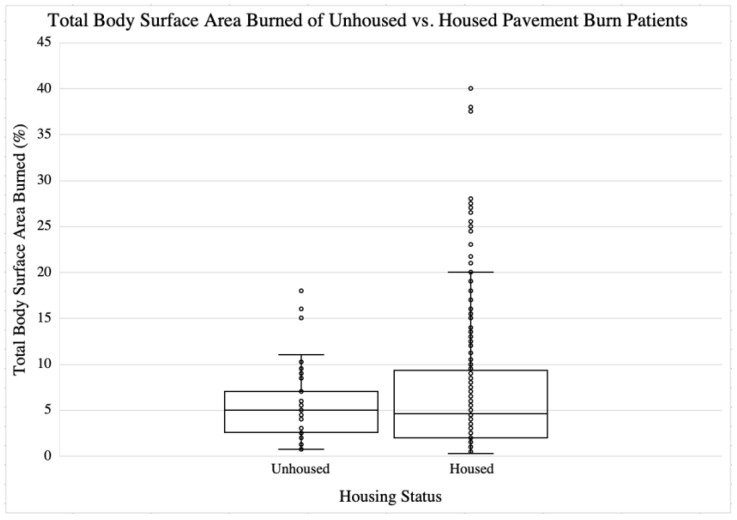
Total body surface area burned for unhoused vs. housed pavement burn patients.

The primary outcome of this study was the difference in TBSA between the patient groups. It was found that there was no significant difference in TBSA between unhoused and housed patients (unhoused = 5.81% vs. housed = 6.81%; 95%CI = (−0.90,0.96); *p* = NS. For the secondary outcomes, it was identified that there was no significant difference in hospital length of stay (unhoused = 22.7 days vs. housed = 19.1 days; [95% CI = −11.5, 4.4]); *p* = NS. The burn injury characteristics and hospital course of the study cohort patients are further depicted in Table 2. A graph of TBSA in unhoused versus housed patients in this study is shown in Figure 2.

### 3.3. Mortality of Unhoused vs. Housed Patients

The results of the logistic regression analysis employed were calculated to demonstrate if there was an association between housing status (unhoused vs. housed) and mortality. After adjusting for age, the odds of mortality for unhoused individuals were (OR 0.24 ± 0.26, 95% CI: 0.03–1.91) compared with housed individuals. However, this result was found to be not statistically significant (*p* = 0.179). The mortality data is shown in Table 3.

## 4. Discussion

The primary aim of this study was to investigate the impact of pavement burns on unhoused patients compared to housed pavement burn patients. This was performed by examining key clinical outcomes, including TBSA, mortality rates, and hospital length of stay. Through an analysis of these factors, this study sought to better elucidate how housing status may correlate with the experience and medical outcomes of pavement burn patients. However, our findings ultimately did not indicate a significant difference in TBSA between unhoused and housed patients (Table 2). Similarly, both the mortality rates and length of stay demonstrated no significant disparities between the two experimental groups (Table 2 and Table 3). These results warrant further investigation and cautious interpretation, particularly in light of the well-documented vulnerabilities of unhoused individuals, and specifically their increased exposure to risk factors for pavement burns emphasized in previous research studies—such as prolonged exposure to outdoor environments, an inaccessibility to medical care, and higher incidence of conditions which may lead to a loss of consciousness [4,6,7,9,10,11].

With regard to our study’s primary outcome measure, TBSA, there was found to be no significant difference between the unhoused and housed patients included in this study (Table 2). This finding notably contradicts previous studies that have suggested that unhoused patients tend to present with more severe burn injuries, including higher TBSA values, potentially due to delayed access to emergency care or prolonged contact with hot surfaces [15,16]. Yet, it is important to consider that there are multiple potential confounding variables that may have impacted the findings of our study. One possible explanation for this discrepancy in our results compared with other studies may be the effectiveness of the emergency response systems and burn care in Las Vegas, the setting where this study was conducted. It is possible that rapid emergency medical service response and the proximity of burn centers to the burn injuries may have mitigated the severity of burn injuries in both housed and unhoused patients, thereby minimizing the potential differences. Unhoused populations have been shown to concentrate in urban areas, and the city in which this study takes place is Las Vegas. Las Vegas is a highly urban city, and there is a specifically high hospital concentration in the urban sectors of the city [18]. This geographic proximity may play a crucial component in reducing the time to care and initial burn injury management. Additionally, the significantly lower mean BMI that was identified in unhoused individuals in this study may have contributed to a lower propensity for larger TBSA burns, as higher BMI has previously been associated with more severe burn injuries due to a larger surface area of skin being exposed (Table 1) [19]. It is also possible that unhoused individuals in Las Vegas have developed more adaptive behaviors over time to avoid prolonged contact with hot surfaces given their increased exposure to the extreme climate, which may have reduced the extent of burn injuries in this cohort. In addition to these potential confounding factors, other studies have also found results aligning with our study [13,20]. For example, one 2024 study found that when comparing burn incidence between patients of different SES in Las Vegas, Nevada, there was no significance of overall SES in regard to burn incidence [13]. This study did, however, identify a high proportion of contact and pavement burns in the most disadvantaged group [13]. Another study conducted in 2024 identified that the TBSA in unhoused vs. housed burn patients was found to not be significantly different [20]. It is notable that this study did state that unhoused populations were associated with a higher incidence of third-degree burns specifically, and these burn injuries were more so related to self-harm and assault—emphasizing the public health variables that exacerbate unhoused individuals’ risk of burn injuries [20].

In regard to the secondary outcome measures, our study similarly found no significant difference in survival to hospital discharge or length of stay between unhoused and housed patients (Table 2 and Table 3). Similarly to TBSA, these findings are unexpected and run counter to several previously published articles that have suggested worse outcomes for unhoused burn patients due to delayed presentation and treatment, as well as known biases in healthcare accessibility [5,12,14]. One 2015 study described that pavement burns are significantly worse than similarly sized burns of other etiologies, including scald and flame burns, with regard to length of stay among other outcome variables [5]. Another 2024 study corroborated this by also finding that pavement burns on average had the longest length of hospitalization when compared with other burn injuries [13]. This same study also found that pavement burn patients were less likely to be discharged home compared with other burn etiologies, such as chemical burns [13]. Finally, another study demonstrated no difference in mortality between housed and unhoused burn patients, but a significantly longer hospital length of stay in unhoused individuals [16]. Other studies have shown that SES is significantly related to mortality rates in burn injuries [21]. It is possible that the single burn center utilized for this study provides equitable care to both unhoused and housed patients, which may have mitigated significant differences in the hospital length of stay and mortality rates for patient groups. However, as previously described in relation to TBSA, it is also likely that components of the study design may have influenced the significance level of these results. Furthermore, this study only looks at a pavement burn etiology for burn injuries in unhoused populations, and it may be possible that broadening the scope to assess other etiologies of burn injury may elucidate a significant difference in unhoused vs. housed burn patient hospital course and outcomes [6]. Yet, other possible explanations in regard to the lack of significance include improved awareness and medical interventions in recent years contributing to more equitable care for unhoused individuals, as well as burn unit protocol implementation designed to handle vulnerable populations, ensuring that unhoused patients receive the same level of care as their housed counterparts.

Despite the findings of our study, it is essential to acknowledge that unhoused individuals continue to remain at a disproportionately high risk for sustaining pavement burns due to their increased exposure to extreme temperatures and significant lack of protective measures [3,16,20,22,23]. The ultimate purpose of this study is to emphasize the importance of implementing resources to prevent burn injuries. Yet, there are various projects that have implemented or proposed public health interventions targeting decreasing pavement burn incidence or ameliorating burn disparities. One study of specific note discussed the creation of a fire safety manual and training sessions for unhoused encampment residents to improve education and prevention [24]. In a 2023 study, researchers identified that tent fires were found to be common among unhoused individuals in the colder months, and they provided equipment and developed escape strategies with these populations that would allow for the avoidance of burn injuries [24]. Although not regarding pavement burns specifically, interventions such as those outlined throughout this study may provide insight into effective strategies for preventing other etiologies of burn injuries in unhoused patients as well. The current interventions to prevent pavement burns among unhoused populations include infrastructure improvements such as shaded areas and cooling stations [25]. Outreach programs through community health initiatives aimed at educating unhoused individuals about the dangers of hot pavements and providing them with protective gear, such as footwear and clothing, may play an integral role in regions such as the Southwestern United States in promoting health equity for unhoused populations [26,27]. It is also important to note that a substantial proportion of the pavement burn injuries found in this study occurred in housed individuals—underscoring the broader public health ramifications of extreme heat. For this population, awareness campaigns on the risk of walking barefoot, hydration, and overall sun exposure may help mitigate the occurrence of preventable pavement burn injuries. Implementing education for prehospital teams may also improve the initial identification of pavement burn injuries, and prevent further damage from laying injured patients on pavements during hot weather [28].

In addition to these community-level interventions, these findings support the need for proactive policy interventions at the local and state level aimed at reducing the incidence of these largely preventable injuries in vulnerable populations. Effective strategies may include expanding accessibility to daytime cooling infrastructure during extreme heat events as well as increasing the availability of shaded infrastructure in dense urban sectors of cities like Las Vegas. Further city planning in regions with a dry climate should also consider heat-reflective pavement coatings or alternative materials in areas with high pedestrian traffic of individuals experiencing homelessness. Beyond this, from a healthcare systems perspective, implementing increased housing assistance resources and social work services in burn as well as emergency care settings may be beneficial in addressing the underlying social determinants of health that impact these injuries.

The strengths of this study include the inclusion of a relatively large cohort of 305 patients in relation to other studies examining similar topics, as well as the study focus being a specific and understudied area of burn injuries. The assessment of pavement burns specifically provides valuable insights into a unique public health issue. Yet it must be noted that there are also multiple limitations to this study that may have substantially affected the results. First, due to the inherent limitations of the retrospective nature of this study, a cause-and-effect relationship cannot be established. Additionally, the relatively small number of unhoused patients in the study (*n* = 54) compared with housed individuals affects the power to detect smaller, but clinically significant, differences. The smaller unhoused population size included in this study does increase the likelihood of committing a type 2 error. Another limitation is that this study relies on data from a single geographic area—Las Vegas, Nevada—which may not be generalizable or adequately represent other regions with different climates, healthcare systems, and unhoused population dynamics. In addition, the potential for underreporting and the misclassification of unhoused status may have significantly affected the accuracy of the findings. Future research in this subject area should take a multi-center approach or prioritize studies with larger and more diverse populations on an international scale to enhance the generalizability of the findings and determine how other demographic factors may affect pavement burn incidence and outcomes. Additionally, employing a prospective study design could allow for more accurate and timely data collection, while simultaneously strengthening causal inferences. Such research should also aim to analyze additional contributing variables, such as comorbid conditions and access to medical care, or injury characteristics including burn depth and complications, that may further influence the incidence, severity, and outcomes of pavement burn injuries across different demographic populations.

## 5. Conclusions

Ultimately, our study found no significant differences in the TBSA, mortality rates, or length of stay between unhoused and housed pavement burn patients. The fact that we did not detect a difference in the meaningful outcomes at our institution is reassuring, and we believe it may reflect consistent care regardless of housing status. We suspect that this pattern is similar to other burn centers who strive to maintain a high level of consistent burn care for all patients. However, the high risk of pavement burn injuries among unhoused individuals underscores the need for continued preventive measures and public health interventions to protect this especially vulnerable population. Demonstrating the disproportionately harmful impact of pavement burns on unhoused populations may provide evidence that increased public infrastructure to provide shade for unhoused individuals in geographic areas prone to an increased incidence of pavement burns may be beneficial in decreasing pavement burn hospitalizations and complications in these communities. Further research with larger sample sizes and prospective designs are needed to confirm these findings and explore additional factors that may influence the outcomes of pavement burn injuries in unhoused populations. Addressing these gaps in knowledge can lead to more targeted interventions and policies aimed at reducing the incidence and severity of pavement burns among unhoused individuals, ultimately improving these patients’ health and quality of life.

## Figures and Tables

**Table 1:** Demographic characteristics of study cohort.

Demographic Characteristics and Logistic Regression ^1^					
Unhoused vs. Housed					
**Housing Status**		***n* (%)**			
Unhoused		54 (18.70%)			
Housed		251 (82.30%)			
Total		305 (100%)			
Age					
**Housing Status**		**Mean ± SD**	**OR**	**CI**	***p*** **-Value**
Unhoused		51.80 ± 12.12			
Housed		55.69 ± 17.69	1.01	0.99–1.03	0.14
Total		55.00 ± 16.88			
Sex					
**Housing Status**		***n* (%)**	**OR**	**CI**	***p*** **-Value**
Unhoused	Female	4 (7.41%)			
	Male	38 (70.37%)			
	Other/Unknown	12 (22.22%)	3.80	1.27–11.32	0.0019 *
Housed	Female	48 (19.12%)			
	Male	111 (44.22%)			
	Other/Unknown	92 (36.65%)			
BMI					
**Housing Status**		**Mean ± SD**	**OR**	**CI**	***p*** **-Value**
Unhoused		24.4 ± 3.7			
Housed		28.8 ± 8.0	1.14	1.06–1.22	<0.0001 *
Total		28.0 ± 7.6			

^1^ BMI, Body Mass Index; OR, Odds Ratio; CI, Confidence Interval; SD, Standard Deviation. * Statistically significant with *p*-value < 0.05.

**Table 2:** Injury characteristics and hospital course of study cohort.

Injury Characteristics ^1^			
*t*-Test Results for TBSA: Unhoused vs. Housed			
**Housing Status**	**Mean ± SD**	**95% CIs**	***p*** **-Value**
Unhoused	5.53 ± 4.14	4.34–6.70	0.16
Housed	6.90 ± 6.64	6.03–7.76	
Total	6.65 ± 6.28	5.91–7.39	
*t*-Test Results for Length of Hospital Stay: Unhoused vs. Housed			
**Housing Status**	**Mean ± SD**	**95% CIs**	***p*** **-Value**
Unhoused	22.22 ± 35.54	12.12–32.32	
Housed	19.76 ± 25.57	16.43–23.08	0.57
Total	20.20 ± 27.56	16.95–23.44	

^1^ SD, Standard Deviation; CI, Confidence Interval.

**Table 3:** Mortality of unhoused vs. housed pavement burn patients.

Logistic Regression of Mortality: Unhoused vs. Housed ^1^					
**Housing Status**	**Dead (*n* = 20)**	**Alive (*n* = 260)**	**OR ± SD**	**95% CIs**	***p*-Value**
Unhoused	1	49	0.24 ± 0.25	0.03–1.84	0.171
Housed	19	211			

^1^ SD, Standard Deviation; CI, Confidence Intervals; OR, Odds Ratio.

## Data Availability

The data presented in this study are available on request from the corresponding author.

## Abbreviations

The following abbreviations are used in this manuscript:

TBSA	Total body surface area
ABA	American Burn Association
ICU	Intensive care unit
SD	Standard deviations
CI	Confidence intervals
BMI	Body mass index
